# Supplementary material for: Single-peptide DNA-dependent RNA polymerase homologous to multi-subunit RNA polymerase
Source: Nat Commun. 2017 Jun 6;8:15774. doi: 10.1038/ncomms15774 (PMC5467207; doi:10.1038/ncomms15774)
Supplement: Supplementary Information — Supplementary Figures and Supplementary Tables [file ncomms15774-s1.pdf]

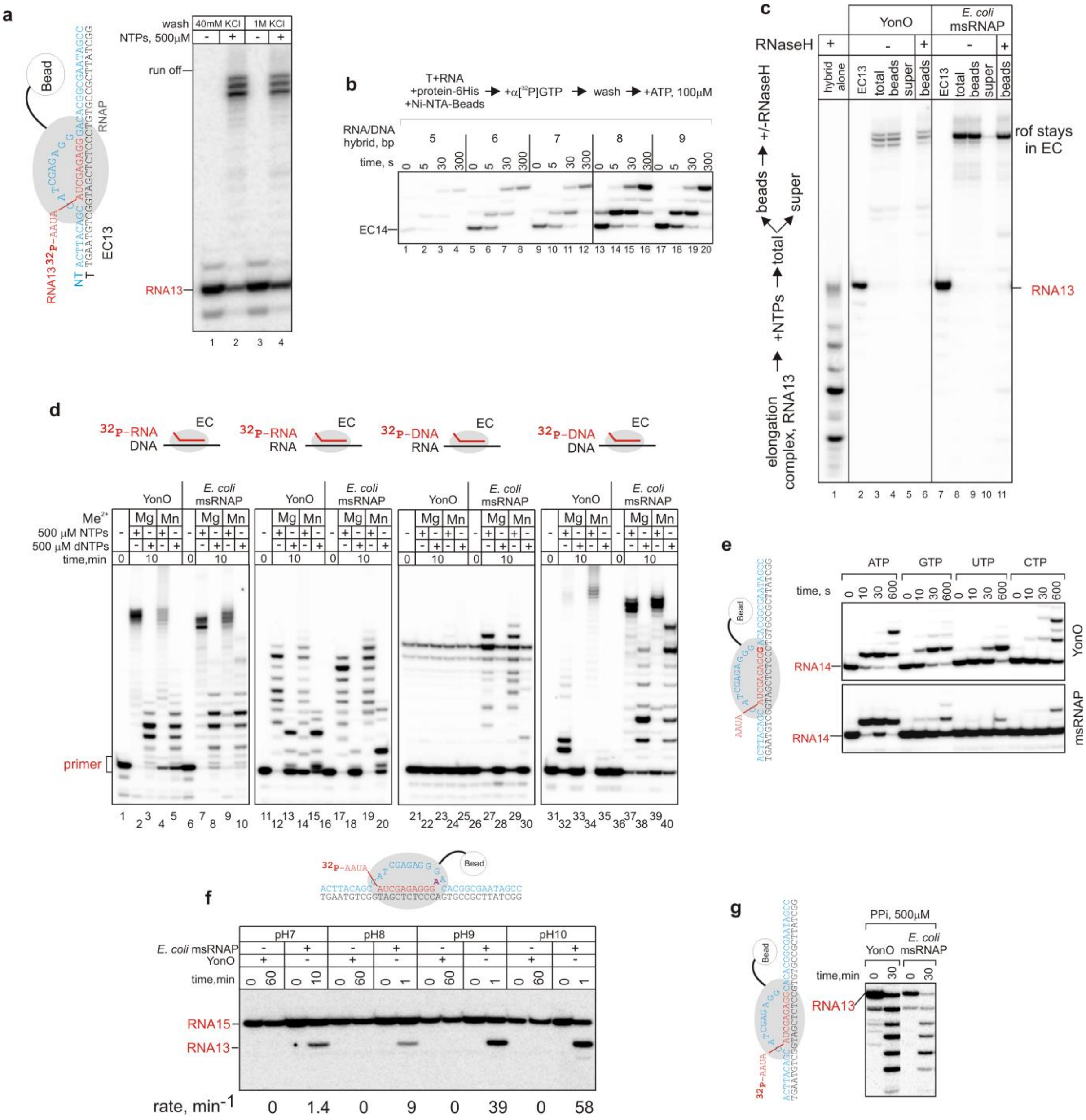

Supplementary Fig. 1. Characteristics of transcription elongation by YonO. a. YonO forms a salt-stable EC. Immobilized ECs were washed with transcription buffer with or without a high salt concentration before NTP addition. b. An 8 bp RNA-DNA hybrid is a prerequisite for stability of EC formed by YonO. The scheme of the reaction is shown next to the gel and sequences of the oligonucleotides are as shown in Table S1. Minor extension at time 0 is due to misincorporation during the RNA labelling with α-[^{32}P]GMP. c. YonO and msRNAP facilitate disengagement of RNA from the template DNA behind the EC. The ECs were chased with NTPs to the end of template. Note that the complexes do not dissociate from the template. RNase H was added to the beads fraction. The scheme of the reaction is shown next to the gel. d. Mn^{2+} has little effect on the specificity of YonO towards template, transcript, or dNTPs (compare to Figure 1d). e. YonO is more error prone than *E. coli* msRNAP (see also Figure 1f). f. YonO cannot hydrolyze phosphodiester bonds of the transcript even at higher pH and prolonged incubation. The apparent rates of the reactions are shown below the gel. g. YonO can perform processive pyrophosphorolysis.

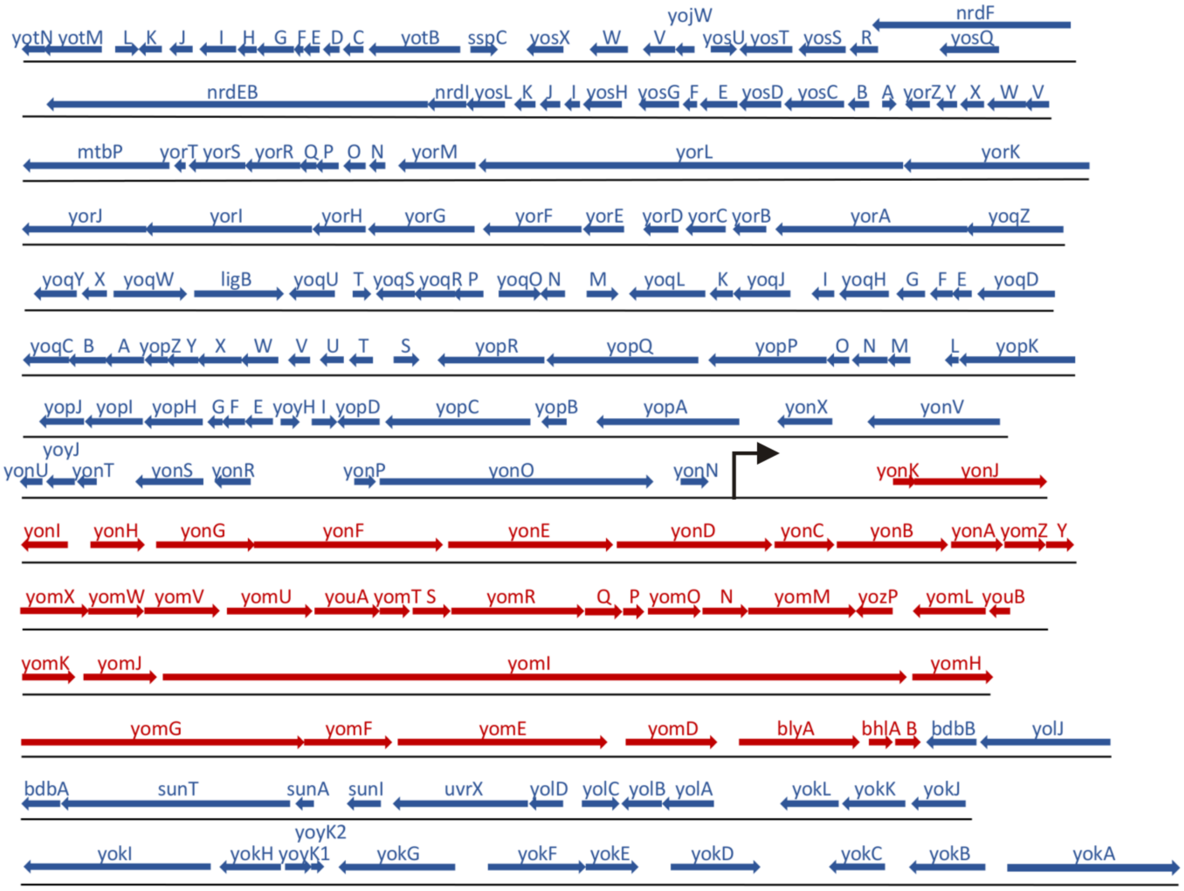

Supplementary Fig. 2. YonO transcribes the late genes of SPβ phage. The map of the SPβ prophage genome. In red are the late genes transcribed by YonO. A black arrow shows the predicted TSS of the YonO transcribed genes.

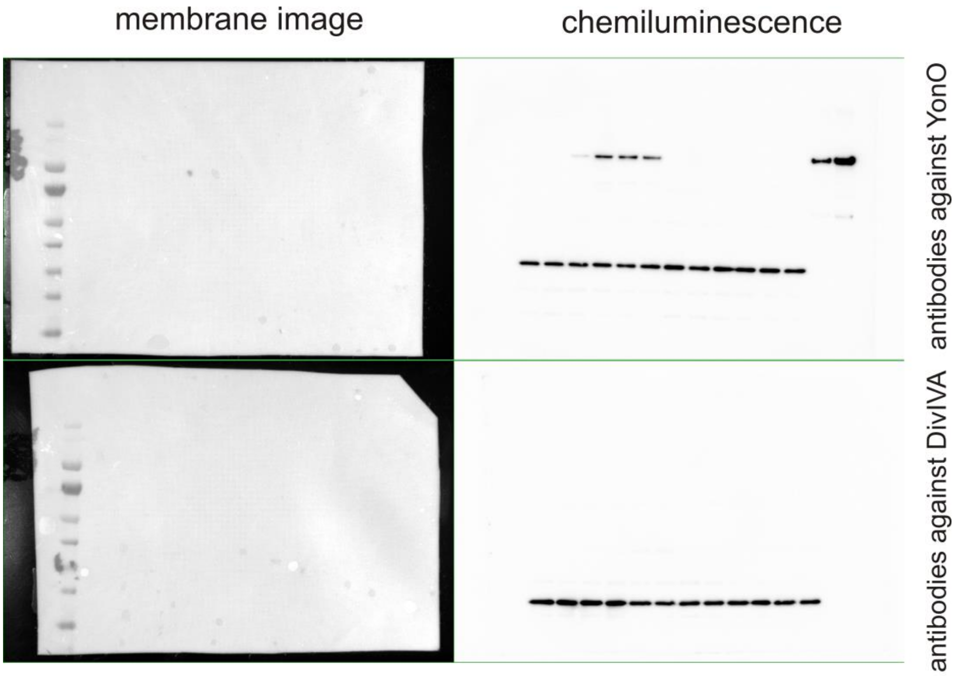

Supplementary Fig. 3. Uncropped western blot shown in Fig. 2a. Left are the images of the membranes with size markers visible. Right are the Western images.

Supplementary Table 1: List of oligonucleotides used in this study.

Primer Name	Sequence (5' → 3')	Purpose
YonO pET28 Fwd	CATATGGCTAGCTTGAAAGGAAAAAAAGACGG	Cloning *yonO* into pET-28a expression vector
YonO pET28 Rev	GTAGTTCTCGAGCTAACGGTGGTTAATTC	
YonO Asp → Asn Fwd	GATATCCAAGCTGCTGCAGTTTAACAATAACGGGAATAAGGCCTTAATTATTTCTGA	Introducing asp to asn substitutions into *yonO* pET-28a plasmid
YonO Asp → Asn Rev	TCAGAAATAATTAAGGCCTTATTCCCGTTATTGTTAAACTGCAGCAGCTTGGATATC	
Δ*yonO* A fragment Fwd	CTGACTACCTTTCAAGGCTGAGATG	Construction of Δ*yonO* MazF cassette for generation of marker-free Δ*yonO* strain
Δ*yonO* A fragment Rev	CAAGGCACTTTTAAATTCTCCCCTTATTAC	
Δ*yonO* B fragment Fwd	GGAGAATTTAAAAGTGCCTTGAGCCTTAC	
Δ*yonO* B fragment Rev	CTGATTGGGTAGGATCCGCGGGTCTTACCGACATGTTTAG	
Δ*yonO* MazF cassette Fwd	CGCGGATCCTACCCAATCAGTACGTTAATTTTG	
Δ*yonO* MazF cassette Rev	CGACAGCGGAATTGACTCAAGC	
Δ*yonO* C fragment Fwd	TTGAGTCAATTCCGCTGTCGTTTGAGTCAGAAAAGCTTTATG	
Δ*yonO* C fragment Rev	GATTCCATAAGCAAGAGTTAGTGCTTC	
Δ*yonO* check Fwd	CCTTAGTTAAAGACAAGTTGC	Confirmation of Δ*yonO* deletion
Δ*yonO* check Rev	GTAAGACTGTGATGGGATTCAC	
P_{YonO} primer extension Fwd	GAATATTAGTGGAGATTCCTCTCC	In vivo primer extension to identify YonO transcription start site
P_{YonO} Primer extension Rev	CTACATAAAAAACTTGAGTAGCAAAGG	
P_{veg} primer extension Fwd	GAACATAATTGAGGAATCATAGAATTTTG	In vivo primer extension of the Pveg promoter transcription start site
P_{veg} primer extension Rev	CGAACGCTCAATCGTTTTTCG	

Supplementary Table 2. Oligonucleotides used for elongation complex assembly. The oligonucleotides are shown according to their orientation and annealing in the assembled elongation complex. Red colouring denotes the position of the ^{32}P label.

Oligonucleotide Sequences	Related Figure
NT-DNA 5′ ACTTACAGCCATCGAGAGGGACACGGCGAATAGCCA 3′<br>RNA 5′ AAUAAUCGAGAGG 3′<br>T-DNA 3′ TGAATGTCGGTAGCTCTCCCTGTGCCGCTTATCGGT 5′	1b, S1a, S1c, S1g
NT-DNA 5′ ACTTACAGCCATCGAGAGGGACACGGCGAATAGCCA 3′<br>RNA 5′ AAUAAUCGAGAGGG 3′<br>T-DNA 3′ TGAATGTCGGTAGCTCTCCCTGTGCCGCTTATCGGT 5′	1c, 1e, S1e
NT-DNA 5′ ACTTACAGCCATCGAGAGGGTCACGGCGAATAGCCA 3′<br>RNA 5′ AAUAAUCGAGAGGGA 3′<br>T-DNA 3′ TGAATGTCGGTAGCTCTCCCAGTGCCGCTTATCGGT 5′	1g, S1f
RNA 5′ AAUAAUCGAGAGG 3′<br>T-DNA 3′ TGAATGTCGGTAGCTCTCCCTGTGCCGCTTATCGGT 5′	1d, S1d
RNA 5′ CCAAAACCUC 3′<br>T-RNA 3′ UUUUUUUUGGAGAGCUAAUAA 5′	
DNA 5′ CCAAAACCTC 3′<br>T-RNA 3′ UUUUUUUUGGAGAGCUAAUAA 5′	
DNA 5′ AATAATCGAGAGG 3′<br>T-DNA 3′ TGAATGTCGGTAGCTCTCCCTGTGCCGCTTATCGGT 5′	
Hybrid Length: 5<br>RNA 5′ AAUAAGGCAGAGG 3′<br>T-DNA 3′ TGAATGTCGGTAGCTCTCCCTGTGCCGCTTATCGGT 5′	S1b
Hybrid Length: 6<br>RNA 5′ AAUAAUGGAGAGG 3′<br>T-DNA 3′ TGAATGTCGGTAGCTCTCCCTGTGCCGCTTATCGGT 5′	
Hybrid Length: 7<br>RNA 5′ AAUACGCGAGAGG 3′<br>T-DNA 3′ TGAATGTCGGTAGCTCTCCCTGTGCCGCTTATCGGT 5′	
Hybrid Length: 8<br>RNA 5′ AAUACUCGAGAGG 3′<br>T-DNA 3′ TGAATGTCGGTAGCTCTCCCTGTGCCGCTTATCGGT 5′	
Hybrid Length: 9<br>RNA 5′ AAUAAUCGAGAGG 3′<br>T-DNA 3′ TGAATGTCGGTAGCTCTCCCTGTGCCGCTTATCGGT 5′	
Hybrid Length: 10<br>RNA 5′ AAGCAUCGAGAGG 3′<br>T-DNA 3′ TGAATGTCGGTAGCTCTCCCTGTGCCGCTTATCGGT 5′	
NT-DNA 5′ ACTTACAGCCATCGAGAGGGCCACGGCGAATAGCCA 3′<br>RNA 5′ AAUAAUCGAGAGGG 3′<br>T-DNA 3′ TGAATGTCGGTAGCTCTCCCGGTGCCGCTTATCGGT 5′	1f
